# Prkg2 regulates alveolar type 2-mediated re-alveolarization

**DOI:** 10.1186/s13287-022-02793-4

**Published:** 2022-03-21

**Authors:** Mo Zhang, Gibran Ali, Satoshi Komatsu, Runzhen Zhao, Hong-Long Ji

**Affiliations:** 1 Department of Cellular and Molecular Biology, University of Texas at Tyler, 11937 US Highway 271, Tyler, TX 75708-3154 USA; 2 Xinxiang Medical University, Xinxiang, China; 3 Texas Lung Injury Institute, University of Texas at Tyler, Tyler, TX USA

**Keywords:** *Prkg2*, Proliferation, Differentiation, Organoids, Lung injury

## Abstract

**Background:**

The cGMP-dependent type 2 protein kinase, encoded by the *prkg2* gene, is highly expressed in alveolar type 2 epithelial (AT2) cells. It is unclear whether *prkg2* regulates AT2 cell homeostasis and re-alveolarization of injured lungs. This study aimed to investigate the role of *prkg2* in the regulation of the fate of AT2 in vitro.

**Methods:**

Primary AT2 cells of wild-type (wt) and *prkg2*^−/−^ mice were co-cultured with fibroblasts as three-dimensional organoids. The colony formation was analyzed between days 4 and 12 post-seeding. EdU assay was used to detect cells with active DNA synthesis. AT1 and AT2 cells in organoids were visualized with anti-podoplanin and anti-surfactant protein C antibodies, respectively.

**Results:**

*Prkg2*^−/−^ AT2 cells developed a greater number of organoids than wt controls. However, compared to wt organoids, a lower number of AT2 but a greater number of AT1 cells were visualized. In addition, a lower number of proliferated cells (EdU^+^) were observed in *prkg2*^−/−^ organoids compared to wt controls. The numbers of organoids and EdU^+^ cells were significantly reduced in protein kinase A (PKA) inhibitor H89-treated wt and *prkg2*^−/−^ cultures. Organoids and EdU^+^ cells were increased by lipopolysaccharides (LPS) in both wt and *prkg2*^−/−^ groups. The increase in the proportion of AT1 and AT2 cells in organoids was only seen in wt controls.

**Conclusions:**

*Prkg2* may regulate the lineage of AT2 cells, which is affected by endotoxins and the interactive PKA signaling pathway.

## Background

The cGMP-dependent protein kinase 2, a member of the serine/threonine kinase family, is encoded by the *prkg2* gene [1]. The *prkg2* gene is highly expressed in the brain, intestinal mucosa, and lungs, and plays an essential role in the regulation of endochondral ossification and proliferation of gastrointestinal and nervous cells [1–6]. Previous studies have shown that *prkg2* is highly expressed in lung epithelial cells, especially in alveolar type 2 (AT2) cells [2]. During lung development, a high expression level of *prkg2* has also been observed in differentiated AT2 cells [7]. Although *prkg2* has been shown to inhibit the growth of lung cancer cells [8], its role in the homeostasis of alveolar epithelial cells is unknown. Given that *prkg2* deficiency causes dwarfism in mice with small lungs [5], *prkg2* may have a role in the renewal of AT2 cells and differentiation into AT1 cells in adult lungs.

Human AT2 cells occupy 2–5% of the alveolar surface area and are a heterogenous population of cells involved in secretory and regenerative roles to maintain lung homeostasis [9]. In normal lung tissues, AT2 cells secrete surfactant proteins to maintain the alveolar tone for gas exchange [10]. During embryonic development, both AT2 and AT1 cells are differentiated from bipotential low columnar progenitor cells present at the distal airway tips and expanding into sac-like configurations [11]. In the alveoli, only AT2 cells have preserved differentiation potential. AT2 cells self-renew and differentiate into AT1 cells to repair and maintain the integrity of alveoli [12].

In the lung epithelial cells, apical ENaC proteins transport salt and fluid to prevent flooding of the air spaces. Using the NO/cGMP/PRKG signaling pathway, *prkg2* signals regulate transepithelial fluid and salt re-absorption through the lung epithelial sodium channels (ENaC) [13–15]. Activation of *prkg2* activates the ENaC to increase alveolar fluid clearance and lung regeneration [14, 16]. *Prkg2* deficiency leads to ENaC dysfunction in mice lungs acutely injured by lipopolysaccharides (LPS) [14]. However, the role of LPS in the regulation of the fate of AT2 by *prkg2* is unknown.

The *prkg2* gene can phosphorylate and activate the cystic fibrosis transmembrane conductance regulator (CFTR), which, combined with environmental changes and aging, permanently affects the responses of AT2 cells [17–19]. *Prkg2* interacts with the protein kinase A (PKA) signaling pathway, which is important for proliferation and CFTR activity [20]. To examine the role of *prkg2* in the regeneration of normal and injured lungs, we compared the lineage of AT2 cells between wt and *prkg2* knockout mice both in the absence and presence of PKA inhibition and endotoxin.

## Methods

### Animal husbandry

The *prkg2*^−/−^ mice were gifted by Dr. Pfeifer’s lab and maintained in the C57 BL/6 background [5]. The mice were raised in pathogen-free conditions. The study protocol was approved by the Institute of Animal Care and Use Committee (IACUC) at the animal facility of the University of Texas at Tyler Health Science Center, Tyler, Texas. The mice were kept in a 12-h light/dark cycle, with free access to food and water. Age- and sex-matched (2–4-month-old, 20–30 g) wild-type (wt) and *prkg2*^−/−^ mice were killed for the experiments in accordance with the IACUC approved procedures.

### Mouse AT2 isolation

AT2 cells were isolated as described previously [21]. Briefly, mice were euthanized and exsanguinated, followed by lung perfusion with 10–20 mL of DPBS (Gibco, Grand Island, NY, USA). The trachea was cannulated, and 1.5–2.0 mL of dispase II (50 units/mL, 354235, Corning Inc., Corning, NY, USA), followed by 0.5 mL of 1% low melting point agarose (A9414, Sigma-Aldrich, St. Louis, MO, USA), was injected into the lungs. The low-melting-point agarose was maintained at 42–45 °C before injection. The lungs were covered with ice for 2 min after injection. Then, the lungs were removed and incubated in dispase II for 45 min at 25 °C. Then, the lungs were gently torn in DMEM/F-12 (D6421, Sigma-Aldrich) + 0.01% DNase (DN25, Sigma-Aldrich) and incubated for 10 min at room temperature. The cell suspension was passed through serial filters (100, 40, 30, and 10 microns; Nitex filters, Corning) and centrifuged at 300×*g* for 10 min at 4 °C. After resuspending the cells in 10 mL of isolation medium (DMEM/F-12 + 10% FBS [A31605-01, Gibco] + 1% Pen/Strep [Gibco]), the cells were incubated with biotinylated antibodies: rat anti-mouse CD16/32 (553143, BD Biosciences, San Jose, CA, USA), rat anti-mouse CD45 (553078, BD Biosciences), and rat anti-mouse Ter-119 (553672, BD Biosciences) for 30 min at 37 °C in a shaking incubator (60 r/min). Cells were pelleted down and incubated with washed streptavidin-coated magnetic particles (10 mg/mL, 65601, Invitrogen, Carlsbad, CA, USA) for 30 min at room temperature. After magnetic-activated negative selection of undesired cells, the pelleted cells were resuspended in 10 mL of the isolation medium. The suspended cells were incubated on a plastic culture dish for 30 min at 37 °C and 5% CO_2_ to remove fibroblasts and then transferred onto plates pre-coated with 1 mg/mL of IgG (I5381, Sigma-Aldrich) for 2 h in a 5% CO_2_ incubator to remove macrophages. The plate was swirled, and unattached cells were centrifuged for 10 min at 300×*g* at 4 °C. Viability was assessed using trypan blue exclusion assay. Purity was confirmed using PAP stain and anti-SFTPC immunofluorescent staining. Only isolated AT2 cell samples with purity > 90% were used for 3D organoid cultures [21].

### Organoid culture

The 3D organoid cultures were performed as described previously [21]. Freshly isolated AT2 cells (6000/transwell) were mixed with MLg2908 cells (2 × 10^5^/transwell, ATCC CCL-206™, USA) and suspended in a 1:1 mixture of growth factor reduced Matrigel (BD Biosciences) and organoid medium (DMEM/F12 + 2 mM l-Glutamine [MP Biomedicals Europe, Illkirch, France] + 10% Active FBS [A31605-01, Gibco] + 1% ITS [41400-045, Gibco] + 1% Pen/Strep and 10 μM TGF beta inhibitor [Ab120163, Abcam, Cambridge, UK]). The cell mixture was seeded on top of polyester membrane cell culture transwell inserts at 45 μL/transwell (Costar 3470: 0.4 μm pore size, 0.33 cm^2^ area, Corning) and incubated for 30 min to allow the matrix to solidify. We added 600 µL of organoid medium to the bottom of the transwell and replaced half (300 μL) of the medium on alternate days. Cultures were grown at 37 °C and 5% CO_2_ for 4–12 days. Then, 10 µM of PKA inhibitor H89 (371963, Millipore, Bedford, MA, USA) was dissolved in the culture medium and applied to cells. For LPS treatment, 1 µg/mL of LPS (L6529, Sigma) was dissolved in the culture medium and applied. H89 and LPS were added to the medium on alternate days.

### Colony-forming assay

As previously described [21], differential interference contrast (DIC) images were captured at 4× using an Olympus IX73 inverted microscope (Olympus, Tokyo, Japan) and C11440 digital camera controlled by HCImagelive software (Hamamatsu, Japan). The number of colonies and colony-forming efficiency were recorded for each group.

### Immunofluorescence of AT2 organotypic cultures for AT1 and AT2 cells

Cultures were washed with PBS and fixed by adding 4% PFA on the apical side for 10 min at room temperature. The 3D structures were permeabilized with 0.2% Triton X-100. Then, the cultures were blocked with 1% BSA and 4% normal goat serum before incubating with anti-SFTPC antibody (1:500, PA5-71680, ThermoFisher Scientific, Waltham, MA, USA) and anti-PDPN antibody (3 μg/mL, MA5-16113, ThermoFisher Scientific) overnight at 4 °C. After washing with PBS three times, the secondary antibodies goat anti-rabbit Alexa Flour 488 (1:500, ThermoFisher Scientific) and goat anti-hamster Flour 568 (1:500, ThermoFisher Scientific) were used to detect the primary antibodies. The organoid embedded membranes were removed and mounted on glass slides using VECTASHIELD mounting medium (with nuclei stain) and glass coverslips, as described previously [21]. Z-stacked images were obtained using a Zeiss LSM 510 confocal microscope (Carl Zeiss Meditec AG, Jena, Germany) and analyzed with Fiji software, an extension of the ImageJ software. Organoids were scanned for Z-sections using optimal width from top to bottom. Images were stacked separately for PDPN and SFTPC to count the cells positive for both markers. The proportion of PDPN- and SFTPC-positive cells was analyzed. For the 3D structure, Z-sections were stacked from top to bottom and saved as video files.

### Quantification of cells with active DNA synthesis

Active DNA synthesis in organoids was determined by the presence of 5-ethynyl-2′-deoxyuridine (EdU), as described previously [21]. Organoids were stained with Click-iT™ EdU Alexa Flour 488 Imaging Kit, according to the manufacturer’s instructions (C10337, Invitrogen). Images from the different experimental groups were analyzed for the percentage of EdU^+^ cells.

### Statistical analysis

The experiments were repeated on four or more animals (*n* ≥ 4 animals/experiment, ≥ 10 organoids/transwell) based on the evaluation of the power of sample size. Cells were only counted between days 8 and 12 to avoid a complicated analysis of complex organoid structures. Data were normally distributed, and the differences in measured variables between the experimental and control groups were evaluated using two-tailed Student’s *t*-test. One-way ANOVA was used for the comparison of more than two groups. Data were expressed as mean ± SEM and considered statistically significant at *p* < 0.05. OriginPro 8.5 was used for statistical analysis.

## Results

### *Prkg2* deficiency improves organoid formation and maturation

**Fig. 1:**
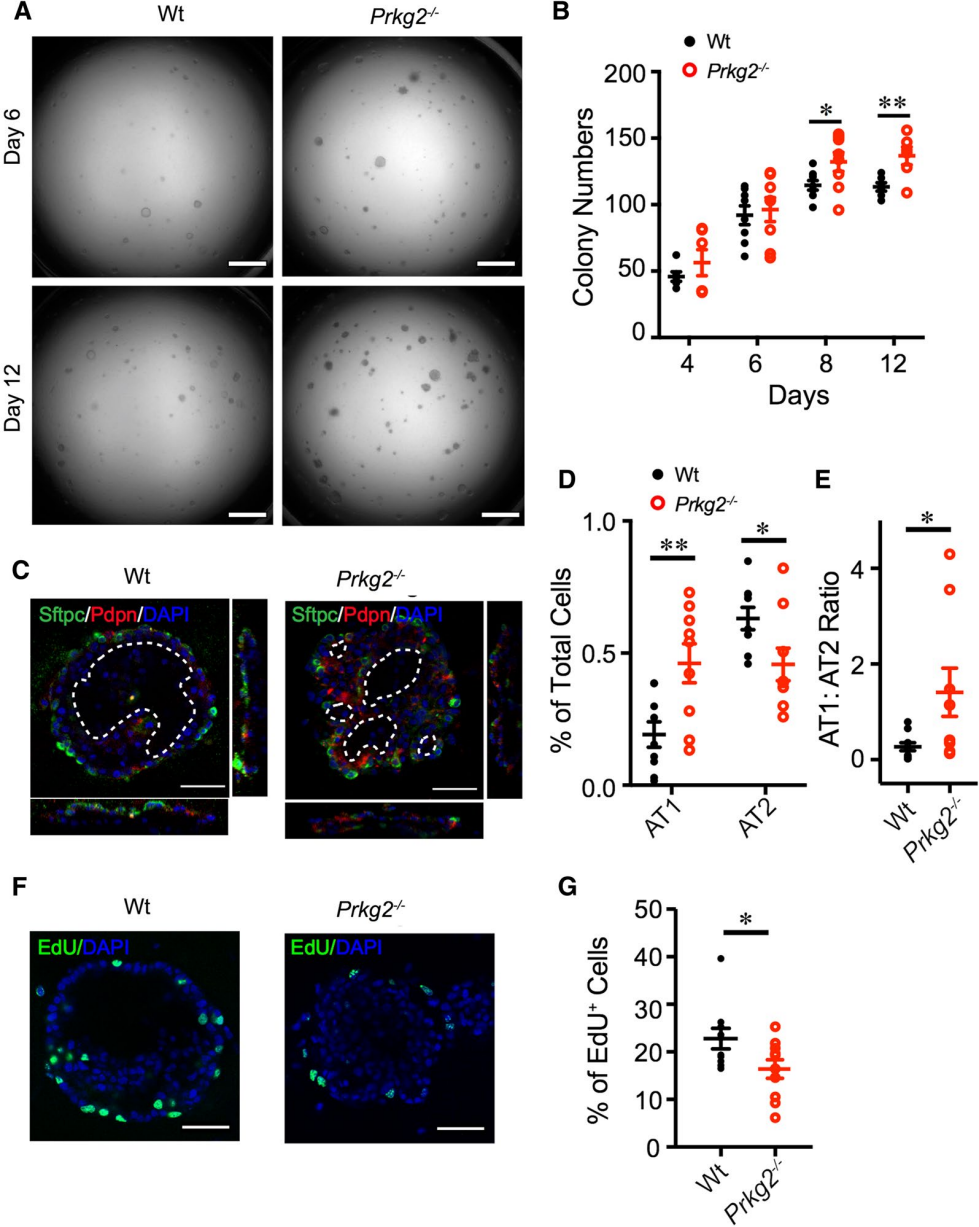
Regulation of the lineage of AT2 cells by *prkg2* in 3D organoids. **A** Differential interference contrast (DIC) images of AT2 organoids on days 6 and 12. Scale bar, 1 mm. **B** Statistical analysis of colony number count of wt and *prkg2*^−/−^ AT2 between days 4 and 12 (*n* = 6 wells, two wells per mouse, three mice per group). **C** Representative images of immunofluorescence stained AT2 organoids on day 12. AT1 (red) and AT2 (green) cells were stained with PDPN and SFTPC antibodies, respectively. Nuclei were counterstained with Hoechst (blue). Dashed white lines indicate the “air space” of spheroids. Scale bar, 50 μm. **D** AT1 and AT2 cell counts (*n* = 10 organoids, 1–2 organoids per well, two wells per mouse, three mice per group). **E** AT1:AT2 ratio (*n* = 10 organoids, 1–2 organoids per well, two wells per mouse, three mice per group). **F** Representative images of EdU^+^ cells. Scale bar, 50 μm. **G** Percentage of EdU^+^ cells (*n* = 10 organoids, 1–2 organoids per well, two wells per mouse, three mice per group). Data are represented as ± SEM, *p < 0.05 and **p < 0.01 vs. wt by two-tailed Student's *t*-test

Given that *prkg2* gene may play a role in lung regeneration, we studied the fate of primary mouse AT2 cells deficient in *prkg2* in 3D organoids. Primary mouse AT2 cells were co-cultured with MLg2908 mouse lung fibroblasts [22]. Organoids of both wt and *prkg2*^−/−^ cultures were visualized between days 4 and 12 (Fig. 1A, B). The number of organoids of *prkg2*^−/−^ cultures gradually increased over the culture time and was significantly greater than that in wt controls on days 8 and 12 (Fig. 1B) (*p* < 0.05). To visualize the 3D structures of organoids, they were immunostained with antibodies against AT1 (PDPN) and AT2 (SFTPC) markers (Fig. 1C). In organoids, AT1 cells formed a “luminal” layer, whereas the outer layer was mainly composed of AT2 cells. Compared to wt controls, multiple small air sac-like hollow spaces were observed in *prkg2*^−/−^ cultures. These results suggest that *prkg2* may be involved in the formation and maturation of AT2 organoids by altering the lineage of AT2 cells.

### *Prkg2* regulates the fate of AT2 cells

Organoid formation and maturation are affected by AT2 lineage. To analyze the lineage of AT2 cells in 3D organoids, we quantified AT1 and AT2 cells for differentiation and proliferation, respectively. In *prkg2*^−/−^ organoids, there were fewer AT2 cells (*p* < 0.01) but more AT1 cells (*p* < 0.05) than those in wt controls (Fig. 1D). Furthermore, the ratio of AT1 and AT2 cells in *prkg2*^−/−^ cultures was significantly greater than that in the wt group (*p* < 0.05) (Fig. 1E). This observation is consistent with the AT1:AT2 ratio reported by others, which is between zero to one in vivo and in vitro [12, 21, 23]. The upregulation of AT2 proliferation by *prkg2* was further confirmed by the EdU assay (Fig. 1F). *Prkg2* deficiency led to a significant decrease in the population of EdU^+^ labeled cells. The percentage of EdU^+^ labeled cells was 16.7% in *prkg2*^−/−^ organoids, which was markedly less than that in wt controls (22.7%, *p* < 0.05) (Fig. 1G). These results suggest that the *prkg2* gene may play a role in the maintenance of AT2 stemness and homeostasis under physiological conditions.

### PKA affects the regulation of AT2 fate by *prkg2*

**Fig. 2:**
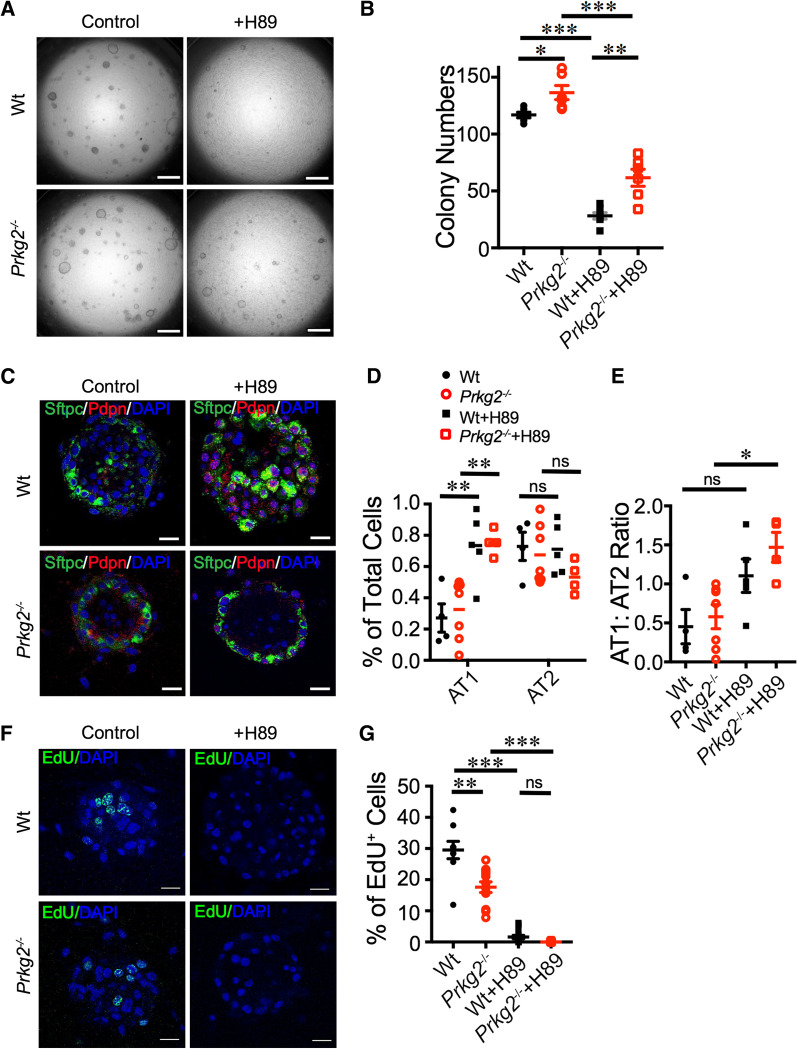
PKA blockade modifies the regulatory effects of *prkg2* on AT2 fate. **A** Representative DIC images of AT2 organoids treated with PKA inhibitor H89 for 8 days. Scale bar, 1 mm. **B** Colony number on day 8 (*n* = 6 wells, two wells per mouse, three mice per group). **C** Representative images of confocal images. Scale bar, 50 μm. **D** Total epithelial cells (*n* = 4 organoids, 1–2 organoids per well, one well per mouse, three mice per group). **E** AT1:AT2 ratio (*n* = 4 organoids, 1–2 organoids per well, one well per mouse, three mice per group). **F** Representative images of EdU-stained organoids. Organoids were labeled with EdU (green) and DAPI (blue). Scale bar, 50 μm. **G** Statistical analysis of the percentage of EdU^+^ cells in organoids (*n* = 10 organoids, 1–2 organoids per well, two wells per mouse, three mice per group). Data are represented as ± SEM, *p < 0.05, **p < 0.01, and ***p < 0.001 vs. controls by two-tailed Student's *t*-test

PKA and PKG signals crosstalk in lung stem cells [24]. The interregulation of both ENaC and CFTR by these two signaling pathways has been reported [2]. In addition, the proliferation of co-cultured fibroblasts in 3D organoids may be regulated by the PKA signaling [25]. To examine the role of PKA signal in AT2 cells in organoids, we added the PKA-specific inhibitor H89 to the culture medium (Fig. 2A)*.* Both *prkg2*^−/−^ and wt groups showed a significant decrease in the number of organoids in the presence of H89 (75.91% reduction for control and 64.05% for *prkg2*^−/−^ organoids, *p* < 0.05) (Fig. 2B). These results suggest a potential interaction between the PKA and *prkg2* signals in the regulation of organoid formation. Furthermore, H89 increased the differentiation of AT1 cells in *prkg2*^−/−^ (*p* < 0.01) and wt organoid (*p* < 0.01) (Fig. 2C, D). The AT1:AT2 ratio was significantly increased in H89-treated *prkg2*^−/−^ organoids (*p* < 0.05) (Fig. 2E). A PKA blockade markedly reduced the EdU^+^ cells in wt cultures, and almost none of the cells were EdU^+^ in *prkg2*^−/−^ organoids (Fig. 2F, G). Based on these observations, PKA-*prkg2* interaction may be involved in the upregulation of AT2 proliferation.

### ***LPS ***did not affect ***prkg2***^−/−^ activated proliferation of epithelial cells

**Fig. 3:**
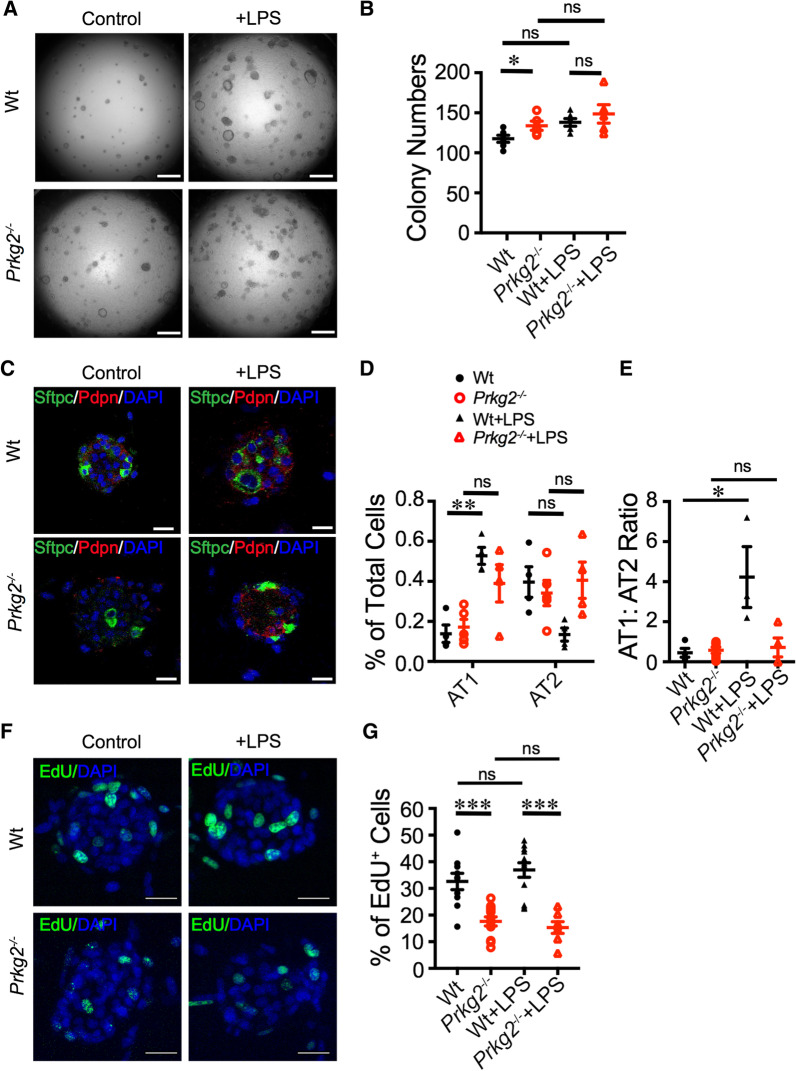
LPS alters the regulation of AT2 lineage by *prkg2* genes. **A** DIC images on day 8. Scale bar, 1 mm. **B** Colony formation on day 8 (*n* = 6 wells, two wells per mouse, three mice per group). **C** Confocal images of AT2 organoids. Scale bar, 50 μm. **D** Epithelial cell count (*n* = 4 organoids, 1–2 organoids per well, one well per mouse, three mice per group). **E** AT1:AT2 cell ratio (n = 4 organoids, 1–2 organoids per well, one well per mouse, three mice per group). **F** Representative images of EdU-stained organoids. Scale bar, 50 μm. **G** Percentage of EdU^+^ cells (*n* = 10 organoids, 1–2 organoids per well, two wells per mouse, three mice per group). Data are represented as ± SEM, **p < 0.05, **p < 0.01, and ***p < 0.001 versus controls by two-tailed Student's *t*-test

Endotoxin is an LPS that constitutes much of the outer membrane of gram-negative bacteria and damages the alveolar epithelium [26]. LPS-induced lung injury is a well-established model of pneumonia caused by microbes. To test the upregulation of the AT2 lineage by *prkg2* gene in injured lungs, we evaluated the proliferation and differentiation of AT2 cells in organoids challenged by LPS. In the wt and *prkg2*^−/−^ cultures, LPS increased the organoids by 17.45% and 12.60%, respectively (Fig. 3A, B), implying an upregulation of AT2-mediated re-alveolarization in LPS-injured lungs. LPS increased AT1 cells (280.37%, *p* < 0.01) but decreased AT2 cells (66.10%, *p* < 0.0001) in wt organoids. However, this regulatory effect disappeared in the *prkg2*^−/−^ group. These observations were further confirmed by the AT1:AT2 ratio. The AT1:AT2 ratio was 4.231 for the wt and 0.718 for the *prkg2*^−/−^ group (*p* < 0.05) (Fig. 3E, F). In addition, LPS caused a mild increase in EdU^+^ cells in wt, but not *prkg2*^−/−^, cultures (Fig. 3G, H). These results indicate that LPS may stimulate AT2 cell differentiation but may also suppress the proliferation of wt AT2 cells in a *prkg2*-dependent manner.

## Discussion

**Fig. 4:**
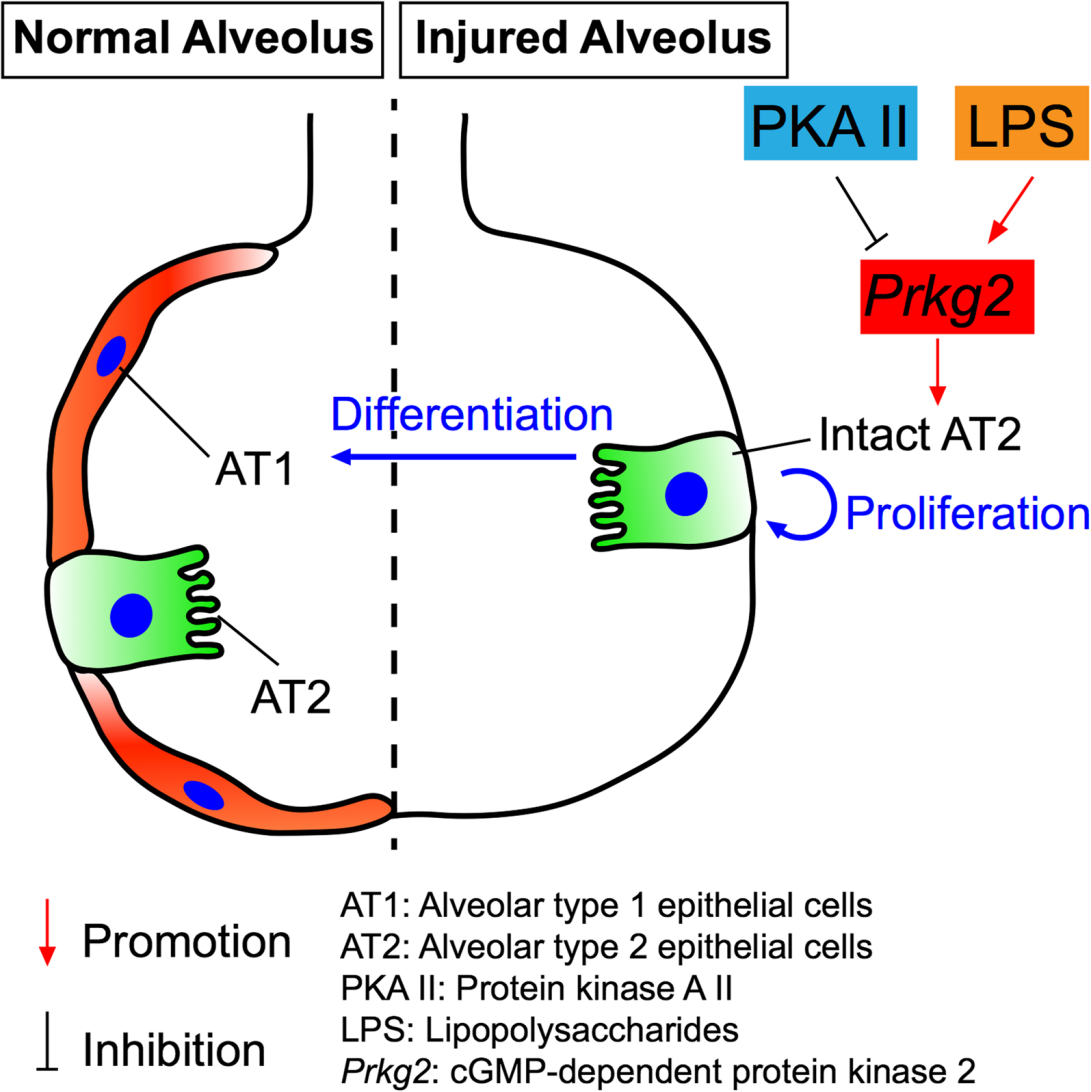
Summary of the role of *prkg2* in regulating the fate of AT2 cells

The aim of the study was to investigate the mechanisms by which *prkg2* regulates the AT2 fate in healthy and injured lungs. *Prkg2* disruption results in a moderate increase in re-alveolarization, which may be due to over-differentiation of *prkg2*^−/−^ AT2 cells, as reflected by the increased AT1 cells and AT1:AT2 ratio. Similar to the inhibitory effect of activated *prkg2* on cell proliferation in non-small cell lung cancer, *prkg2* may down-regulate lung cell proliferation [8]. The PKA inhibitor and LPS exert regulatory effects on the lineage of wt AT2 cells to a greater extent compared to those in the *prkg2*^−/−^ group. These observations indicate that *prkg2* may regulate the homeostasis and re-alveolarization mediated by AT2 stem cells (Fig. 4).

The mechanisms underlying the increased spheroid formation in *prkg2*-deficient AT2 cultures may involve the loss of AT2 stemness and/or exhaustion of proliferating cells. This notion is supported by the appearance of more alveolus-like organoids, differentiated AT1 cells, reduced AT2 cells, elevated AT1:AT2 ratio, and less EdU^+^ cells. The process of organoid development had occurred before our analyses. Because we applied 3D cultures by mixing AT2 cells with fibroblasts and Matrigel [27–29], *prkg2*-expressing feeder cells may interfere with the regulation of AT2 fate in AT2 cells [23, 30–38]. Suppression of *prkg2* is essential for the Wnt/cGMP/Ca^2+^ signaling cascade to regulate the regeneration of lung stem cells [38–40]. Thus, it is possible that the *prkg2*^−/−^ AT2 cells provide feedback to activate the Wnt signaling pathway. Furthermore, NO/cGMP/PRKG2 has been demonstrated to inhibit epidermal growth factor (EGF)-induced MAPK/ERK/JNK-mediated signal transduction in epithelium. One limitation of this study is that the genes regulated by prkg2 deficiency. Multi-omics and sc-seq analyses are required to identify significantly differentiated genes and prioritize regeneration-related signaling pathways.

PKA-*prkg2* interaction may be involved in the regulation of re-alveolarization. PKA may amplify the effects of *prkg2* on the lineage of AT2 cells, as shown by the colony number, AT1:AT2 ratio, and cells with active DNA synthesis. Indeed, in the *prkg2*^−/−^ cultures, the inhibitory effect of the PKA inhibitor on the AT2 fate was significantly reduced. There are two types of cAMP-dependent protein kinase, termed PKA I and PKA II, which are composed of seven isoforms in total. A classic study has demonstrated that only PKA II is involved in proliferation of AT2 cells [41]. Thus, we reason that *prkg2* could interact with PKA II to regulate the lineage of AT2 cells. To confirm this possibility, the mouse line deficient in both *prkg2* and PKA II genes is required. Unfortunately, it is unavailable.

The regulation of the AT2 fate by *prkg2* may depend on endotoxins. Although LPS stimulate the formation of spheroids in wt cultures, this effect was diminished in *prkg2*^−/−^-deficient cells. We postulate that *prkg2* may contribute to the LPS-induced augmentation of re-alveolarization. Alternatively, LPS and *prkg2* may compete for the same mechanism. However, it is unclear how *prkg2* regulate LPS-induced signaling in injured lungs. Further studies are necessary to explore related signaling pathways.

## Conclusions

In summary, our study demonstrated that the *prkg2* gene may regulate the lineage and stemness of AT2 cells to maintain the lung’s hemostasis under physiological conditions. This regulation is affected by the PKA signaling pathway. Suppression of *prkg2* gene may alter the re-alveolarization in infected lungs. This study will inform further exploration of underlying mechanisms for the role of *prkg2* in re-alveolarization.

## Data Availability

All data generated or analyzed during this study are included in this published article.

## Abbreviations

Prkg2: CGMP-dependent protein kinase 2
AT1: Alveolar type 1 epithelial cells
AT2: Alveolar type 2 epithelial cells
3D: Three-dimensional
ENaC: Epithelial sodium channels
PKA: Protein kinase A
LPS: Lipopolysaccharides
